# Intra-aortic Balloon Counterpulsation-Assisted Cardiac Support in the Anesthetic Management of Surgery for Acute Cervical Spine Compression With Severe Coronary Artery Disease in the Prone Position

**DOI:** 10.7759/cureus.117179

**Published:** 2026-09-29

**Authors:** Priya Motiani, Swathi Mallikarjuna, Murali Chakravarthy, Shafiya Shaikh

**Affiliations:** 1 Anaesthesiology, Fortis Hospitals, Bangalore, IND; 2 Anaesthesiology, Fortis Hospital, Bangalore, IND

**Keywords:** cervical spine, coronary artery disease, counterpulsation, elderly, prone position

## Abstract

Severe coronary artery disease associated with the requirement for urgent cervical spine surgery in an elderly patient in a prone position is challenging for the anesthesiologist. Perioperative hemodynamic parameters are key to surgical success. Intra-aortic balloon counterpulsation (IABC) is a well-established modality for the management of patients with ischemic heart disease. There are very few case reports on management in noncardiac surgery with IABC. Here, we present a case report of a 74-year-old male patient with cervical cord compression scheduled for C3-C7 laminectomy and lateral mass fixation, with severe coronary artery disease and a recent history of severe coronary artery disease with triple-vessel disease and severely impaired left ventricular function, managed with IABC insertion post-induction of general anesthesia for intraoperative hemodynamic management to prevent cervical cord hypoperfusion and perioperative myocardial ischemia/infarction.

## Introduction

The simultaneous occurrence of acute coronary syndrome (ACS) and severe cervical spinal cord compression requiring urgent surgical intervention is rare. Prone positioning during cervical spine surgery may significantly reduce venous return and preload, resulting in decreased cardiac output, hypotension, myocardial ischemia, and compromised spinal cord perfusion [1]. These physiological changes pose a substantial risk of myocardial infarction and quadriplegia. Therefore, maintaining hemodynamic stability and preventing perioperative myocardial ischemia are critical for achieving favorable surgical outcomes.

An intra-aortic balloon pump (IABP) is a percutaneous mechanical circulatory support device that is relatively easy to deploy and is associated with a low complication rate [2]. By augmenting diastolic coronary perfusion and reducing left ventricular afterload, intra-aortic balloon counterpulsation (IABC) improves myocardial oxygen supply-demand balance and provides hemodynamic support during periods of cardiovascular stress [3-5]. Its role becomes particularly relevant in patients undergoing surgery in the prone position. We report the successful perioperative use of IABP in a patient with ACS with non-ST-elevation myocardial infarction (NSTEMI), an ejection fraction of 45%, and global hypokinesia of the left ventricle, undergoing cervical spine surgery for acute onset of weakness due to cervical cord compression.

## Case presentation

A 74-year-old gentleman presented to the emergency department with an acute onset of weakness involving both upper and lower limbs, accompanied by exertional chest pain and discomfort. He had a history of a fall at home due to dizziness. ECG showed anterolateral ischemia, and the troponin T level was 313. Echocardiography showed global hypokinesia of the left ventricle and regional wall motion abnormalities, with an ejection fraction of 45%. Coronary angiography demonstrated severe triple-vessel coronary artery disease (Figures 1, 2), and a diagnosis of ACS with NSTEMI was established. The patient was managed medically with heparin 5000 units (injection), a diuretic, and aspirin (tablet). Revascularization was not beneficial as the coronary arteries were thin and completely occluded.

**Figure 1:**
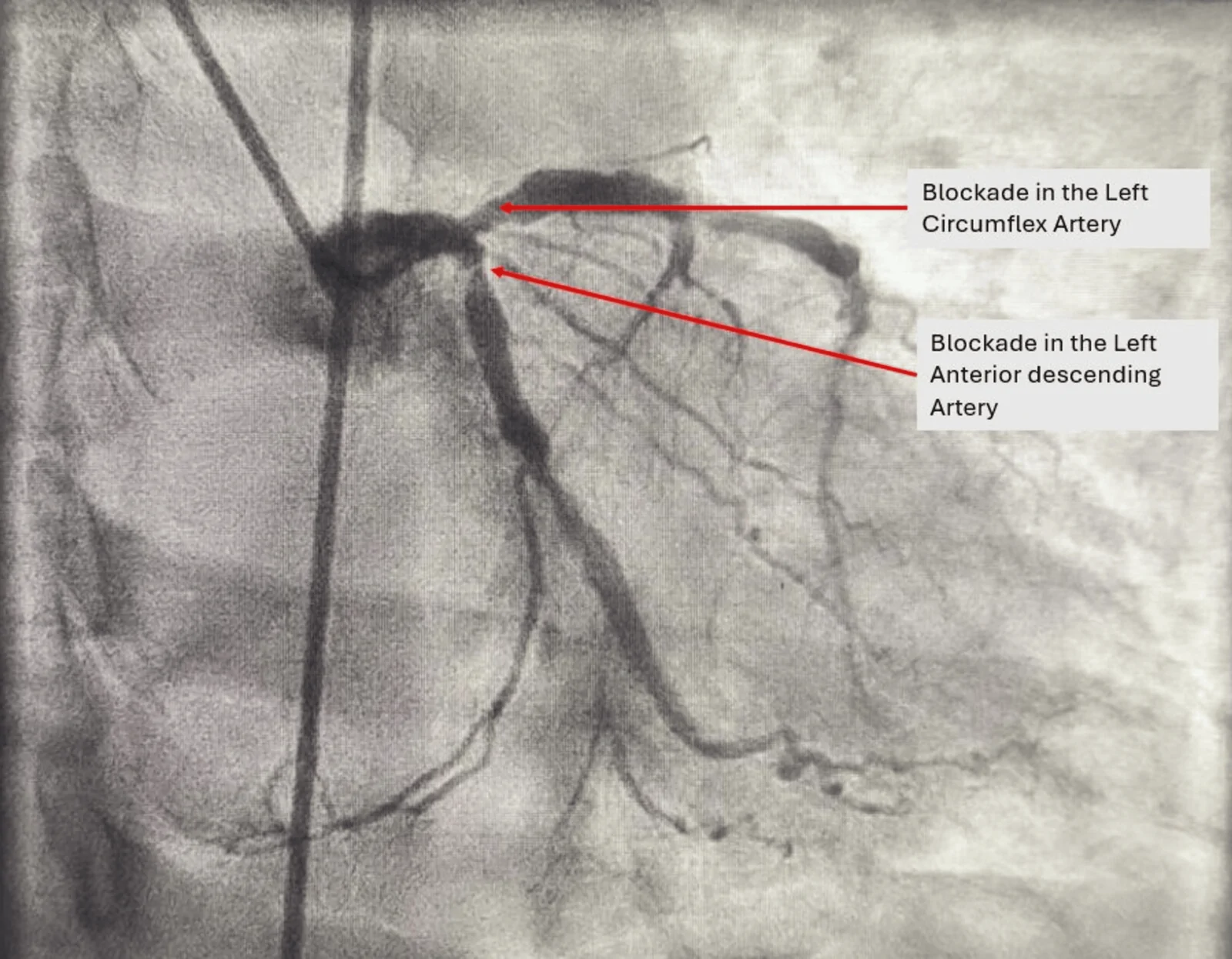
Coronary angiogram showing left coronary artery blockade

**Figure 2:**
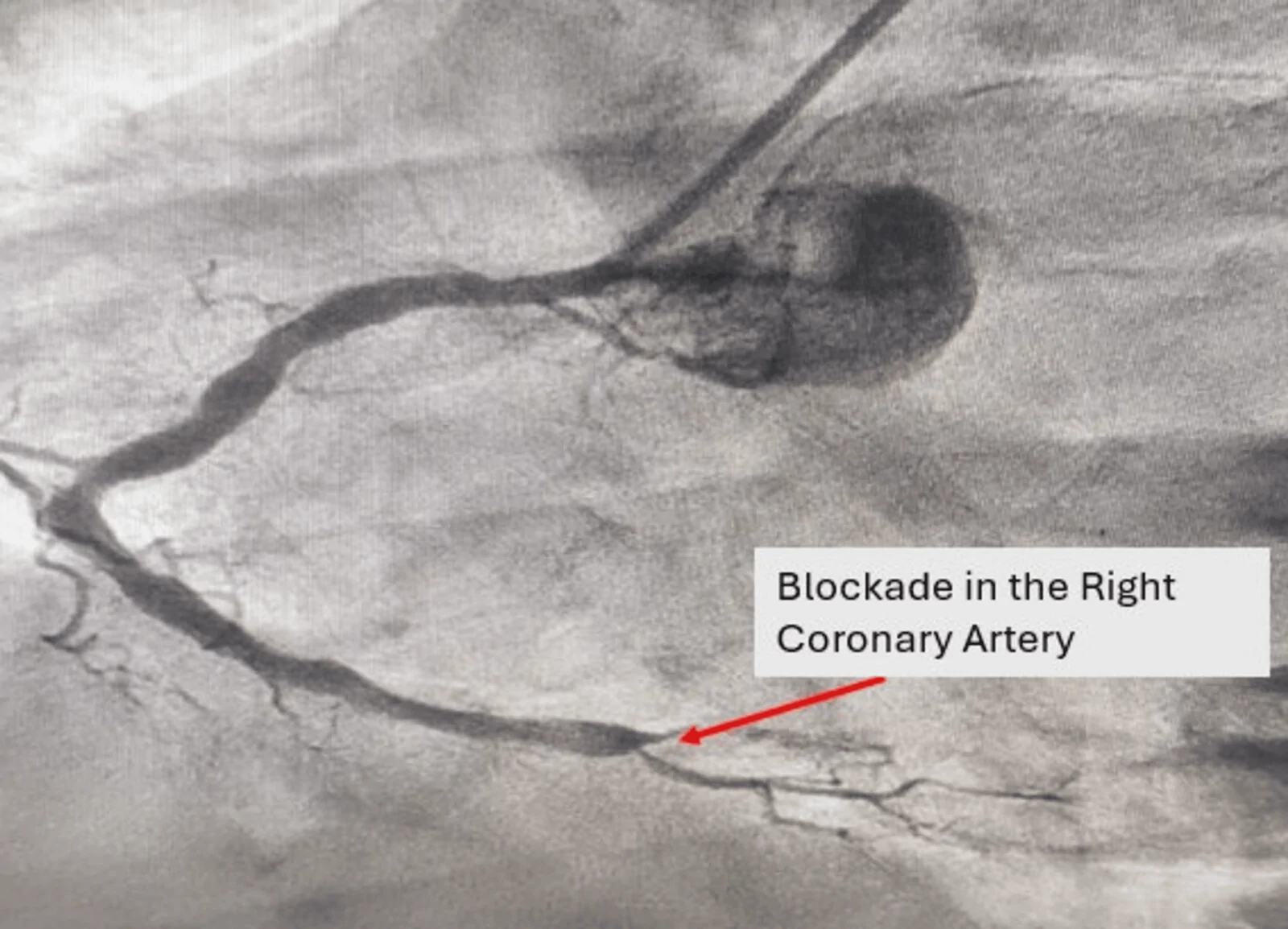
Coronary angiogram showing right coronary artery blockade

Magnetic resonance imaging of the cervical spine revealed severe spinal cord compression from C3 to C7 (Figure 3), necessitating urgent surgical decompression to prevent irreversible neurological deterioration. His medical history was significant for hypertension, diabetes mellitus, and chronic obstructive pulmonary disease, all of which were well controlled with ongoing medical therapy.

Because urgent cervical spine surgery was indicated, coronary revascularization could not be performed beforehand, as postoperative antiplatelet therapy would have complicated the neurosurgical procedure. To prevent further weakness of the upper and lower limbs, we proceeded with cervical spine surgery after medical stabilization for three days. Following institutional anesthesia protocols, standard monitoring was established [6]. The preoperative heart rate was 92 beats per minute (bpm), and blood pressure was 106/54 mmHg. Radial arterial and right internal jugular venous access were secured under local anesthesia. Noradrenaline infusion was started via the central line at a dose of 0.02 mcg/kg/min. General anesthesia was induced with fentanyl (2 μg/kg), etomidate (0.2 mg/kg), and atracurium (0.5 mg/kg). Immediately after induction of anesthesia, there was a drop in blood pressure to 80/48 mmHg, with a heart rate of 80 bpm, which was managed with noradrenaline infusion and a bolus dose of mephentermine 3 mg. As intraoperative neuromonitoring was included, anesthesia was maintained with infusions of fentanyl at 1 mcg/kg/hour, propofol at 50 mcg/kg/min, and ketamine at 0.5 mg/kg/hour after induction of anesthesia.

Again, there was an episode of hypotension to 70/40 mmHg; hence, after induction, an IABP (CARDIOSAVE Hybrid, Getinge AB, Gothenburg, Sweden) was inserted through the right femoral artery. Correct positioning was confirmed using intraoperative transesophageal echocardiography (Figure 4). The patient was then placed in the prone position and underwent C3-C7 laminectomy with lateral mass fixation (Figure 5). Intraoperative hemodynamics remained stable, well within baseline, with a heart rate of 70-80 beats per minute and systolic blood pressure of 90-110 mmHg and diastolic blood pressure of 50-70 mmHg, with a background infusion of noradrenaline at 0.02 mcg/kg/minute and IABP support with an ECG trigger of 1:1 and 100% augmentation throughout the procedure. The propofol, fentanyl, and ketamine infusions were stopped toward the end of the surgery. At the conclusion of surgery, the patient was extubated successfully in the operating room. The patient was shifted to the ICU with IABP at 100% augmentation and noradrenaline infusion at 0.02 mcg/kg/minute. The IABP was gradually weaned by decreasing the augmentation and was removed the next day. The noradrenaline infusion was also tapered and discontinued. Neurological examination revealed improvement in power from 3/5 to 4/5 in both upper and lower limbs and a reduction in tingling sensation in the limbs. Antiplatelet therapy was continued two days after surgery. The remainder of the hospital stay was uneventful, and the patient was discharged with improvement in neurological symptoms.

**Figure 3:**
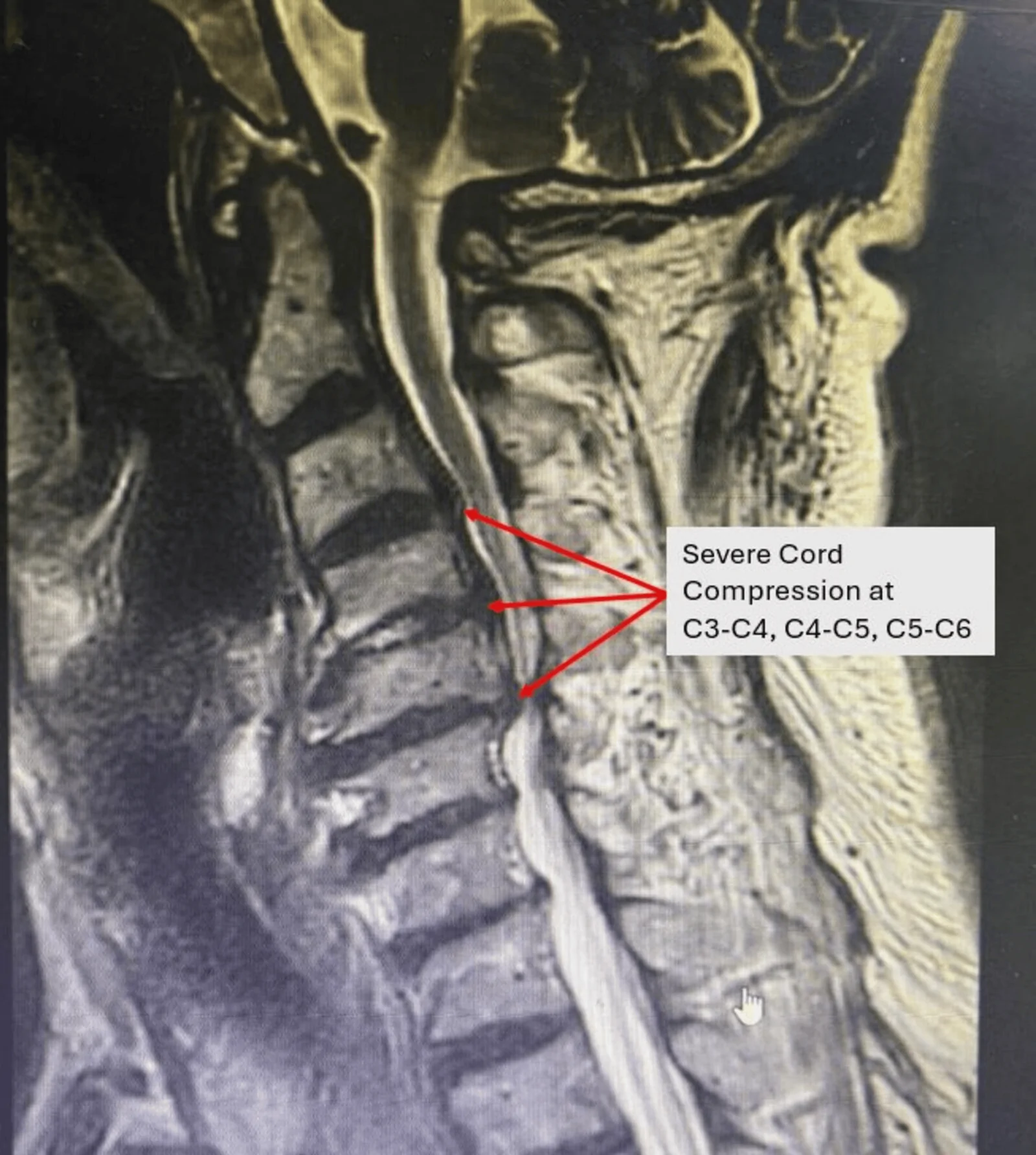
A saggital MRI showing severe cervical cord compression at C3-C6

**Figure 4:**
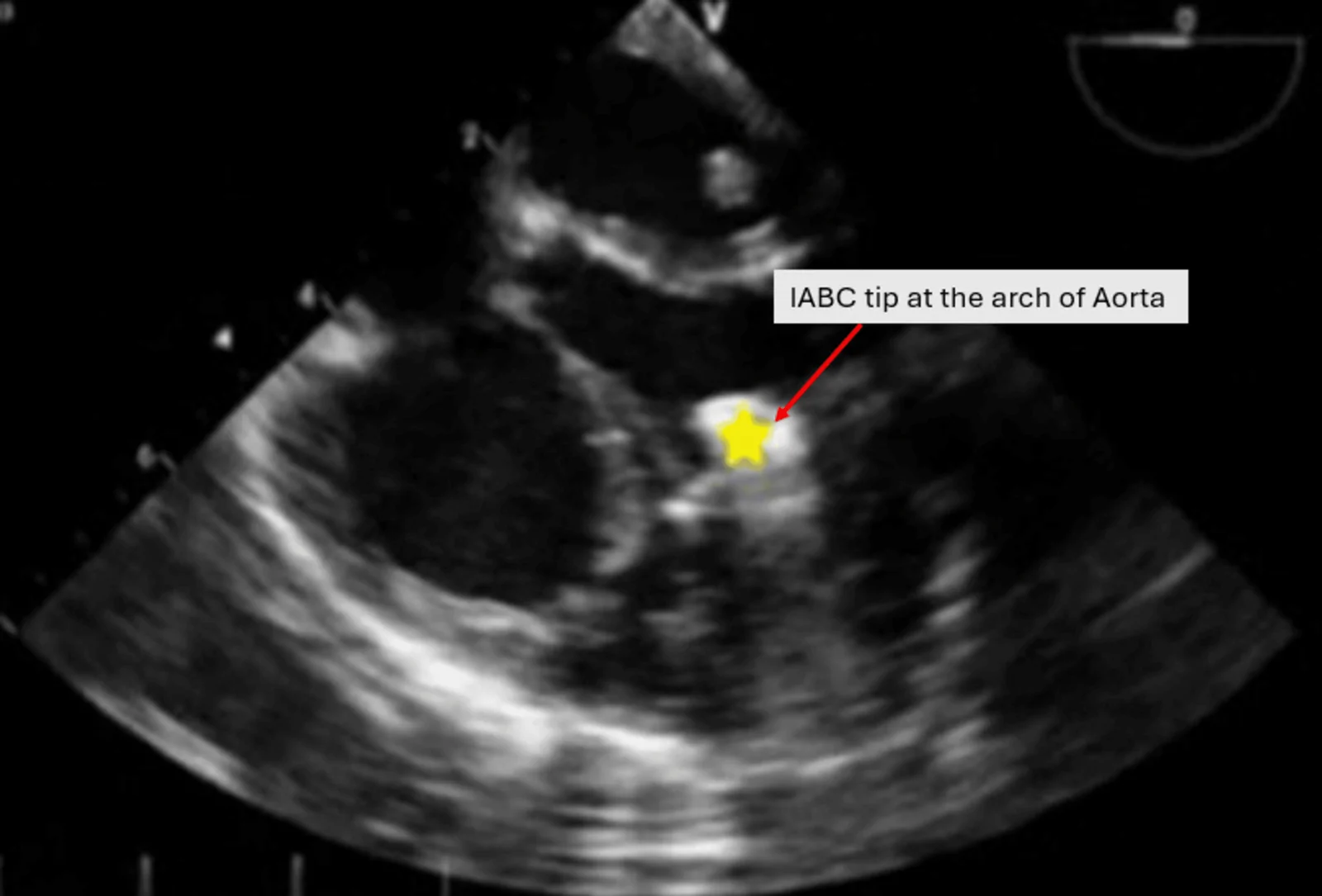
TEE showing the IABP tip in the arch of the aorta IABP: intra-aortic balloon pump, TEE: transesophageal echocardiography.

**Figure 5:**
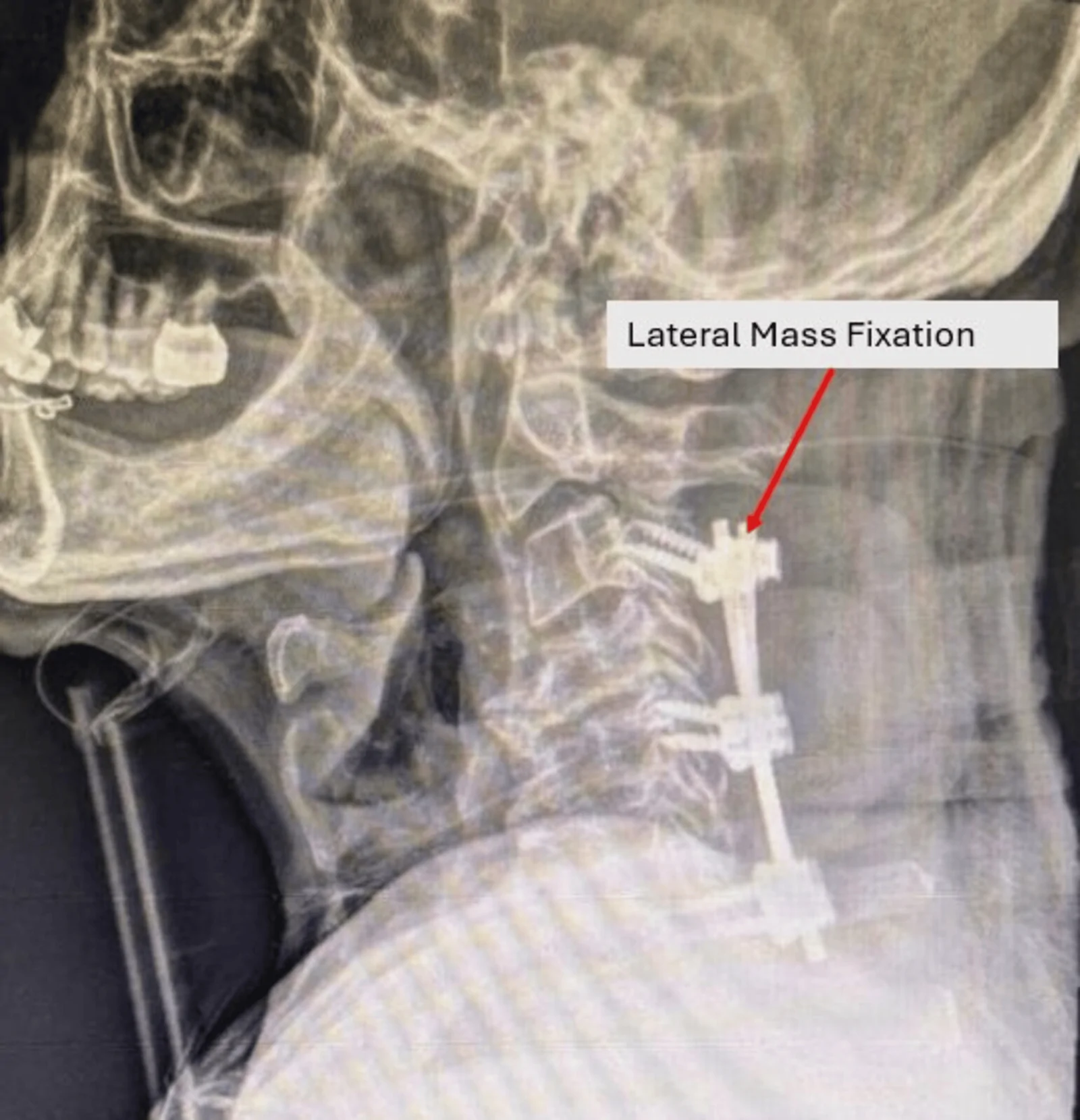
Lateral X-ray post surgery showing cervical fixation

## Discussion

Acute cervical spinal cord compression requiring urgent surgical decompression in a patient with concomitant ACS and severe left ventricular (LV) dysfunction presents a complex perioperative challenge. Cervical spine surgery performed in the prone position presents several perioperative challenges, including hypotension, blood loss, venous thromboembolism, cardiovascular instability, and airway-related complications [7]. The coexistence of a time-sensitive neurological indication and significant myocardial dysfunction creates a situation in which postponement of surgery may result in irreversible neurological deterioration, whereas proceeding with surgery may expose the patient to substantial risk of myocardial ischemia, hemodynamic instability, and perioperative cardiac decompensation. In the present case, the presence of acute cervical cord compression necessitated urgent surgical intervention despite ACS, global hypokinesia of the LV, and associated regional wall-motion abnormalities.

Given the urgency of spinal decompression and the inability to proceed with coronary revascularization before surgery, perioperative management required a strategy that would minimize cardiac risk while enabling timely neurological intervention. The use of an IABP provided temporary mechanical circulatory support and allowed the surgical procedure to be undertaken under conditions of improved hemodynamic stability. The combination of meticulous anesthetic management and IABP support facilitated stable intraoperative hemodynamics and uncomplicated postoperative recovery.

IABC is widely used in patients with myocardial oxygen supply-demand mismatch and provides circulatory support through synchronized counterpulsation. Inflation during diastole increases coronary perfusion pressure and myocardial oxygen delivery, while rapid deflation before systole reduces left ventricular afterload and myocardial oxygen consumption [8,9]. The resulting reduction in LV workload and myocardial oxygen demand, together with improved coronary perfusion, can be particularly useful in selected patients with acute myocardial ischemia and severe LV dysfunction. In the present setting, these effects were clinically relevant because the patient's myocardium had limited contractile reserve and was required to tolerate a major noncardiac surgical procedure. These physiological effects improve cardiac performance and help maintain hemodynamic stability during high-risk surgical procedures.

The decision to proceed with IABP support should be individualized and based on the severity of myocardial dysfunction, extent of ischemia, urgency of the noncardiac procedure, and the availability of alternative mechanical circulatory support. Current practice has become more selective regarding routine IABP use in cardiogenic shock and acute myocardial infarction because randomized trials have not demonstrated a universal mortality benefit from routine IABP therapy. However, this does not necessarily preclude its use as a temporary hemodynamic support strategy in carefully selected patients with severe LV dysfunction who require urgent surgery. In the present case, the objective was not definitive treatment of ACS by IABP, but rather optimization of myocardial oxygen balance and circulatory support during a high-risk noncardiac surgical procedure.

Only a limited number of reports have described the use of IABP in patients with severe coronary artery disease undergoing noncardiac surgery [10,11]. The present case illustrates the potential role of a multidisciplinary strategy in managing patients in whom an urgent neurological procedure cannot safely be delayed despite significant cardiovascular disease. The combination of acute cervical cord compression, ACS, global LV hypokinesia, regional wall-motion abnormalities, and the requirement for prone positioning represents an exceptionally high-risk perioperative scenario. In such circumstances, the anesthesiologist must simultaneously maintain coronary perfusion, preserve spinal cord perfusion, prevent further myocardial injury, and provide a safe anesthetic for surgery. Mechanical circulatory support with IABP may serve as a bridge through this critical period in appropriately selected patients.

## Conclusions

The increasing number of elderly patients undergoing cervical spine surgery has led to more frequent encounters with significant cardiovascular comorbidities. In patients with severe coronary artery disease requiring urgent cervical decompression in the prone position, perioperative IABP support may provide effective hemodynamic stabilization and reduce the risk of myocardial ischemia. This case highlights the value of mechanical circulatory support as a bridging hemodynamic strategy when neurological urgency and severe cardiovascular compromise coexist. Importantly, this experience should not be interpreted as evidence that IABP should be routinely used for noncardiac surgery in patients with ACS or LV dysfunction. Though trials and studies on the perioperative use of IABP are lacking, in selected patients, it may provide a bridging strategy when urgent surgery is unavoidable. The decision should be individualized following multidisciplinary assessment of the risks of delaying neurological decompression against the cardiovascular risks of proceeding with surgery.
